# Feasibility, Usability, and Safety of ParaGym, an Intelligent Mobile Exercise App for Individuals With Paraplegia: Protocol for a Pilot Block-Randomized Controlled Trial

**DOI:** 10.2196/45652

**Published:** 2023-05-19

**Authors:** Janika Bolz, Adrian Löscher, Rainer Muhl, Andreas Badke, Hans-Georg Predel, Claudio Perret

**Affiliations:** 1 Institute of Cardiovascular Research and Sports Medicine German Sport University Cologne Cologne Germany; 2 BG Clinic Tübingen Tübingen Germany; 3 Swiss Paraplegic Research Nottwil Switzerland; 4 Faculty of Health Sciences and Medicine University of Lucerne Lucerne Switzerland

**Keywords:** spinal cord injury, mixed methods, exercise, fitness, home-based, algorithm, personalization, prototype, telemedicine, digital, mobile app, mobile phone

## Abstract

**Background:**

Exercise is crucial for individuals with paraplegia to reduce the risk of secondary diseases and improve independence and quality of life. However, numerous barriers such as inadequate accessibility restrict their participation in exercise programs. Digital exercise apps can help overcome these barriers. Personalization is considered a crucial feature of mobile exercise apps, as people with paraplegia have individual requirements regarding exercise programs depending on their level of impairment. Despite the increasing popularity of mobile exercise apps, there are none available that target the individual needs of this cohort. The ParaGym mobile exercise app prototype was designed to automatically tailor exercise sessions to the individual needs of users with paraplegia.

**Objective:**

This study aims to evaluate the feasibility, usability, safety, and preliminary effectiveness of the ParaGym mobile exercise app prototype.

**Methods:**

This pilot block-randomized controlled feasibility trial will include 45 adult participants with paraplegia. Eligible participants will be block randomized to either the intervention or waitlist control group. The intervention group will perform a 6-week exercise program using the ParaGym mobile exercise app, comprising three 35-minute exercise sessions per week. The waitlist control group will continue their usual care and receive access to the app after study completion. Participants will record all exercise sessions conducted with the app as well as additional exercise sessions conducted during the study period using exercise diaries. The primary outcomes include feasibility, usability, and safety. Feasibility will be assessed through semistructured interviews, study adherence, and retention rates. Usability will be measured using the System Usability Scale. Safety will be determined by the occurrence of adverse events. Secondary outcomes include the effects of the intervention on peak exercise capacity (VO_2_ peak); handgrip strength; independence, which will be measured using the Spinal Cord Independence Measure III (SCIM III); and health-related quality of life, which will be measured using the Short Form–36 Health Survey (SF-36).

**Results:**

Recruitment commenced in November 2022. Overall, 12 participants were enrolled at the time of submission. Data collection commenced in January 2023, with completion expected in April 2023.

**Conclusions:**

To the best of our knowledge, this is the first study to assess the feasibility, usability, and safety of an intelligent mobile exercise app for individuals with paraplegia. Thereafter, the app should be adapted according to the findings of this trial. Future trials with an updated version of the app should aim for a larger sample size, longer intervention duration, and more diverse target group. In the long term, a fully marketable version of the ParaGym app should be implemented. This would increase the access to personalized, independent, and evidence-based exercise training for this cohort and, in the future, other people who use wheelchairs.

**Trial Registration:**

German Clinical Trials Register DRKS00030370; https://drks.de/search/de/trial/DRKS00030370

**International Registered Report Identifier (IRRID):**

DERR1-10.2196/45652

## Introduction

### Background

A high level of sedentary behavior is associated with an increased risk of noncommunicable diseases, such as type 2 diabetes, cardiovascular diseases, and cancer [1]. This especially affects people who depend on a wheelchair for mobility, such as those with a spinal cord injury (SCI) [2,3]. Individuals with SCI show lower physical activity levels than able-bodied individuals and individuals with other chronic diseases [3,4]. This results in a 2- to 4-fold increased risk of obesity, diabetes, and cardiovascular diseases [5]. To prevent comorbidities and secondary diseases resulting from a sedentary lifestyle, exercise is of fundamental importance for this cohort [2,6,7]. Regular exercise increases muscle strength and cardiovascular fitness [8-13], leading to greater independence [10,14], improved mobility [8,14], and higher quality of life [11,15]. Furthermore, individuals with SCI who are engaged in sports show substantially higher social integration [15-17] and fewer depressive symptoms [10,17] than those in the inactive comparison group.

For individuals with SCI, engaging in sports is complicated by multiple infrastructural (eg, inadequate accessibility to buildings), internal (eg, not knowing how to exercise), and monetary barriers [18-20]. Digital exercise platforms have the potential to overcome these barriers and promote exercise participation because they do not require transportation, offer a guided program addressing the needs of individuals with SCI, and may be cheaper than a gym membership or personal training [21]. Using a digital exercise platform has been shown to promote adherence to exercise guidelines in able-bodied individuals during the COVID-19 pandemic, when access to sports facilities was restricted [22]. It can be assumed that this is also applicable to individuals with SCI. However, despite the wide variety [23] and recent increasing popularity of mobile fitness apps [24-26], a mobile exercise app targeting the needs of people with paraplegia does not exist.

Only 2 studies have assessed the feasibility of exercise apps for individuals with SCI, the WHEELS mobile health app [27] and the Fisiofriend app [28], although neither of these is available on the mobile app market. People with SCI have individual requirements regarding exercise programs depending on the level of impairment of their trunk stability and leg function [2,29]. Therefore, the exercise programs provided by apps must be personalized [30]. The exercise programs delivered by these 2 apps can be individualized by the users themselves [27] or by a clinician [28], but untrained individuals may not have the expertise to create and tailor their own exercise programs. In addition, the requirement of support from a health care professional further restricts the user’s independence.

To our knowledge, there is no mobile fitness app available that provides an exercise program for individuals with paraplegia that is automatically tailored to the user’s requirements while the user is completely independent of health care professionals. A prototype of the ParaGym mobile exercise app was developed to enable people with paraplegia to have their own virtual personal fitness coach in addition to their usual care. The app uses an intelligent algorithm that automatically personalizes its exercise program to the user’s needs, considering their physical capacity, fitness level, and exercise equipment.

Bizzarini et al [28] and Hoevenaars et al [27] showed that using a mobile app to promote physical activity in this cohort was feasible. However, there are no instances of conducting an exercise program tailored by an algorithm in this cohort. Therefore, a pilot study is needed to assess whether exercise using the new ParaGym mobile exercise app is feasible and safe for people with thoracic or lumbar SCI.

### Aims of the Study

The primary aims of this trial are as follows:

- To qualitatively assess the feasibility of an app-based exercise program (ParaGym) tailored by an algorithm to the needs of people with paraplegia
- To investigate the usability of the ParaGym app prototype in terms of the app’s design, information provided by the app, app’s ease of use, satisfaction with the app, and app’s functionality using the System Usability Scale (SUS)
- To assess the safety of this program by considering adverse events (AEs) and serious AEs (SAEs)

The secondary aim is to analyze the effects of a 6-week training intervention using the ParaGym mobile exercise app on peak exercise capacity, handgrip strength, independence, and health-related quality of life.

## Methods

### Study Schedule

The SPIRIT (Standard Protocol Items: Recommendations for Interventional Trials) 2013 statement was used to develop the content, quality, and completeness of this protocol [31]. A SPIRIT study schedule (Table 1) and checklist (Multimedia Appendix 1) are provided.

**Table 1:** SPIRIT (Standard Protocol Items: Recommendations for Interventional Trials) 2013 study schedule.

				Study period						
				Enrollment (t_0_)		Allocation (t_0_)		Postallocation period		Postintervention measure (t_1_)
**Enrollment**										
	Eligibility		✓							
	Informed consent		✓							
	Medical screening		✓							
	Allocation				✓					
**Intervention**										
	App						✓			
	Usual care						✓			
**Assessment**										
	**Participant characteristics**									
		Demographics			✓					
		Lesion level			✓					
		Lesion completeness			✓					
		Time since injury			✓					
		Anthropometrics			✓					
	**Feasibility**									
		Exercise diary					✓			
		Adherence					✓			
		Retention					✓		✓	
		Interview							✓	
	**Usability**									
		System Usability Scale							✓	
	**Safety**									
		Adverse events					✓			
		Serious adverse events					✓			
	**Effectiveness**									
		VO_2_ peak^a^			✓				✓	
		Maximal heart rate			✓				✓	
		Resting blood pressure			✓				✓	
		PO peak^b^			✓				✓	
		Borg RPE^c^			✓				✓	
		Handgrip strength			✓				✓	
		SCIM III^d^			✓				✓	
		SF-36^e^			✓				✓	

^a^VO_2_ peak: peak oxygen uptake.

^b^PO peak: peak power output.

^c^Borg RPE: Ratings of Perceived Exertion Scale.

^d^SCIM III: Spinal Cord Independence Measure III.

^e^SF-36: Short Form–36 Health Survey.

### Development of the ParaGym Mobile Exercise App Prototype

The development of the ParaGym mobile exercise app prototype is based on the Kernwerk mobile exercise app (Kernwerk GmbH), which was designed to improve general fitness in able-bodied individuals. The Kernwerk app offers daily functional training sessions created by exercise scientists and uses an intelligent algorithm to individualize these sessions to the user’s needs. The goal of the ParaGym project is to develop a prototype of an intelligent mobile exercise app for individuals with paraplegia.

The ParaGym mobile exercise app uses the same algorithm to provide an automatically individualized training program for people with paraplegia. The steps taken to develop the app are as follows. A user-centered approach was adopted to create the ParaGym exercise app for people with paraplegia. This included a survey among the target group and an extensive review of the literature [32] to identify both health-related and disability-related demands for an exercise program and a mobile fitness app. The results of the survey and the literature review revealed the following: (1) there is an urgent need for accessible and personalized exercise programs; (2) exercises with free weights are preferred over exercises with machines; (3) the exercise pool should contain stretching exercises and exercises to improve scapula coordination; (4) workouts should focus on the dorsal rather than the ventral shoulder musculature to avoid overuse injuries; and (5) users should be provided with comprehensive information about the potential risks of exercising (eg, increased risk of autonomic dysreflexia) and, if required, be cleared by a physician.

On the basis of the results of the survey and literature review, in cooperation with physiotherapists and sports scientists from different spinal cord centers, we developed a pool of 56 exercises to strengthen the core, upper, and lower body musculature; increase aerobic capacity; and improve wheelchair mobility and flexibility. Exercise videos and step-by-step descriptions were added for each exercise. Before integrating the exercises into the app, all exercises, including exercise videos and descriptions, were tested and evaluated in terms of feasibility, usability, and comprehensibility by 10 workshop participants with paraplegia [33]. Adjustments to all 3 components were made based on the feedback of the workshop participants and 3 physiotherapists. This resulted in a total of 67 exercises.

**Figure 1:**
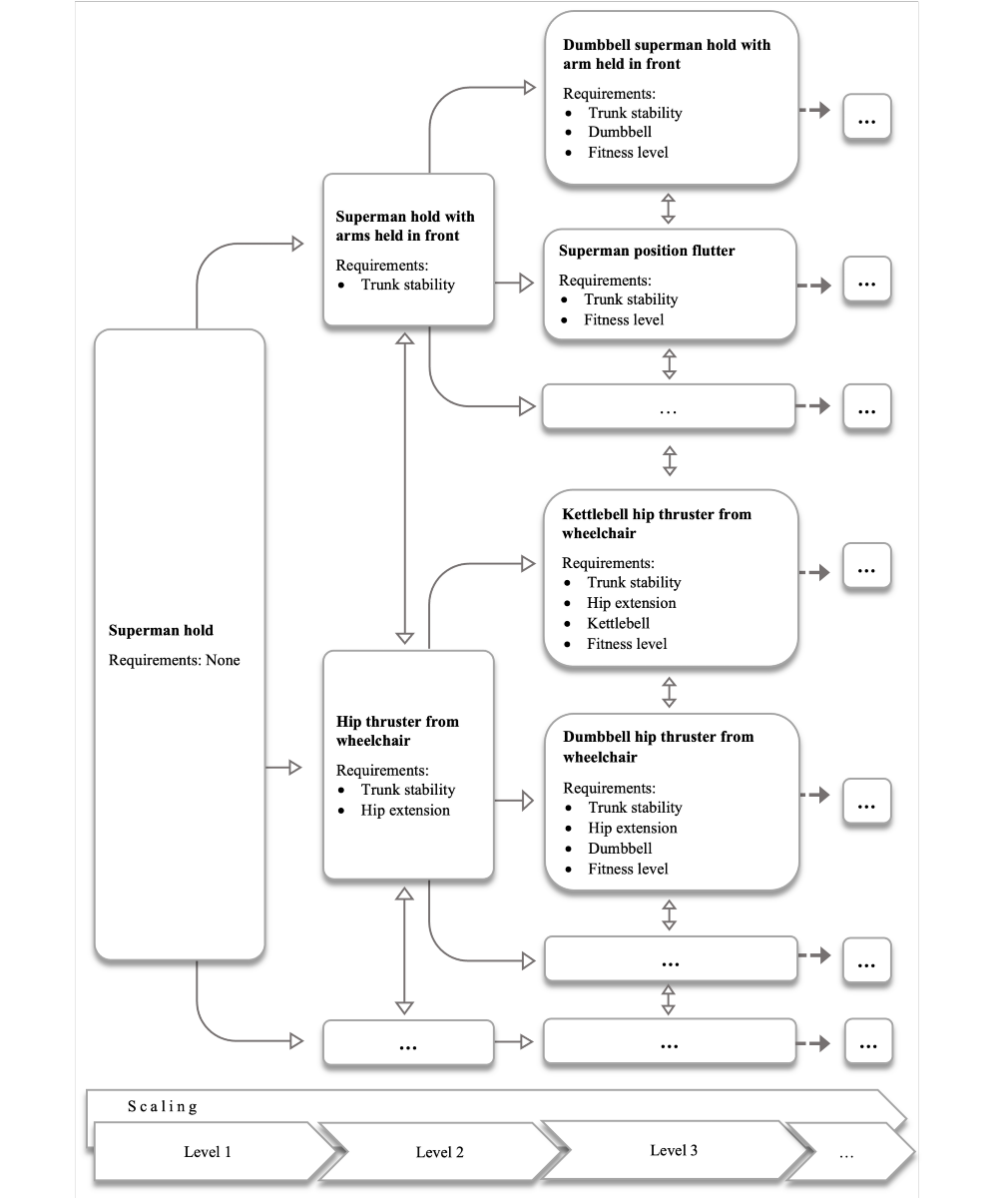
Example of an exercise scaling tree for the exercise “Superman hold.”

**Figure 2:**
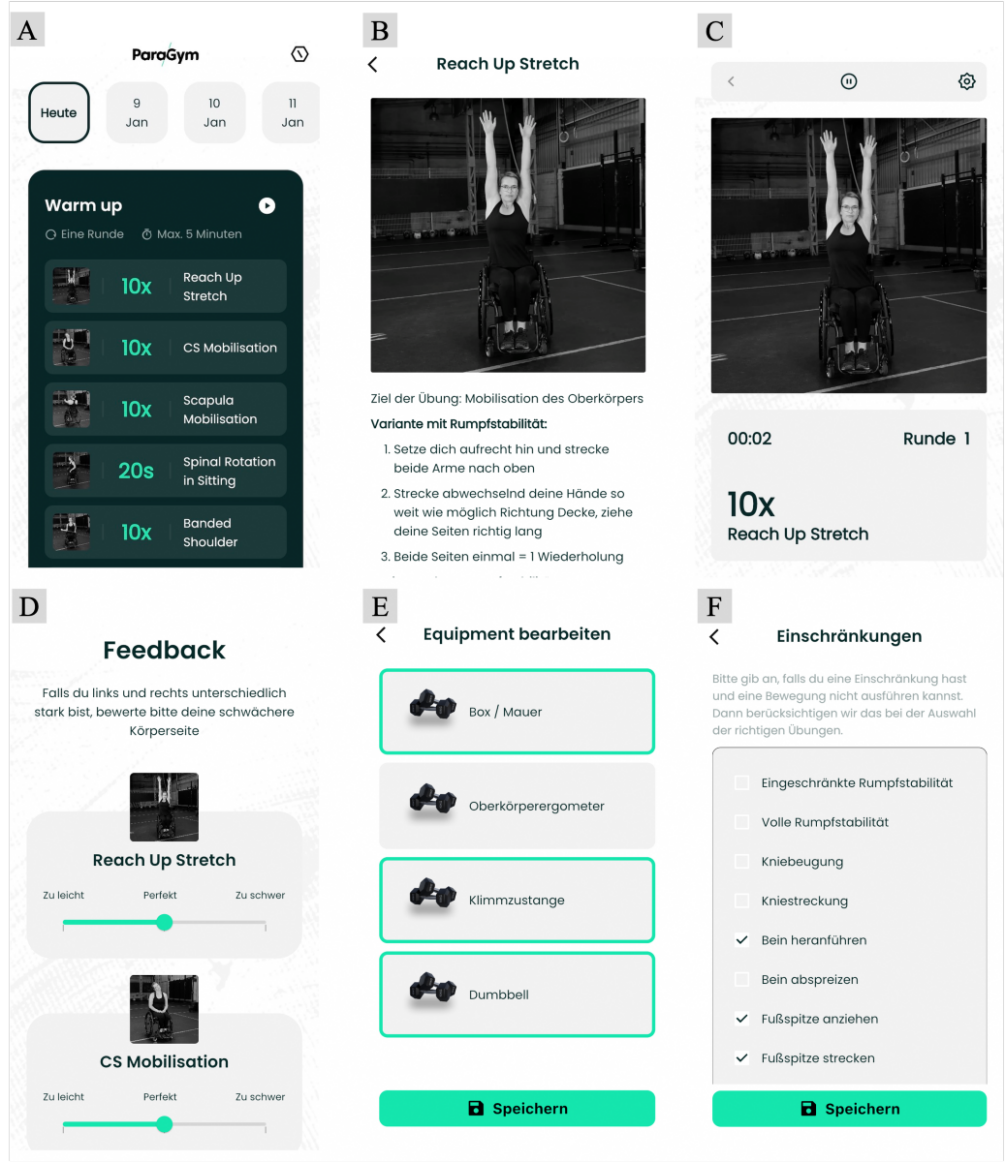
Overview of the ParaGym mobile exercise app prototype. (A) Summary of the daily training session and home screen. (B) Exercise video and description. (C) Screen during workout mode showing the first exercise. (D) Difficulty level rating. (E) Exercise equipment settings. (F) Physical capacity settings.

In the next step, further attributes were added for each exercise, specifying the required equipment (eg, dumbbells), required environmental conditions (eg, sufficient space for wheeling), difficulty level ranging from 1 (very easy, no specific requirements) to 150 (very difficult), and required physical capacity (eg, the extent of core stability and articular movements, such as “hip flexion” or knee extension). In addition, similar exercises were linked to each other. The linkage of the exercises and attributes results in an exercise scaling tree (Figure 1). All these factors are considered by the algorithm so that if 1 attribute does not apply, a similar but more appropriate exercise will be suggested automatically. Whereas information on available training equipment and environmental conditions is provided by the users, the appropriate difficulty level is assessed by rating each exercise on a scale from 1 (too easy) to 7 (too hard) after each training session (Figure 2D). If an exercise is rated “too easy,” a more advanced version of this exercise will be suggested to the user in the following training sessions. Thus, the intelligent algorithm can adjust the exercise level to the current fitness level of the user.

Training sessions focusing on different training goals (eg, improvement of wheelchair mobility or increase in strength) were created based on the exercise pool. The exercises and training sessions were then integrated into the mobile app.

### Description and Content of the ParaGym Mobile Exercise App Prototype

The ParaGym mobile exercise app prototype offers a daily training session that is automatically tailored to the individual requirements of people with paraplegia. Users must input information on their height, weight, age, gender, available exercise equipment (eg, dumbbells), and physical capacity (eg, impaired trunk stability and ability to extend the knee) when registering. The home screen of the app summarizes the daily training session (Figure 2A). Users can choose between different workouts by selecting another date on the timeline above the workout summary. Upon selecting one of the exercises within this summary, the exercise video and description will be displayed (Figure 2B). If one of the exercises selected for the workout is not appropriate, it can be excluded through the settings as described in the subsequent paragraph. To start a workout, users press “play.” Before starting the first exercise, users must confirm that they have read the general safety advice (eg, regarding bowel and bladder management) and specific advice regarding the execution of the exercise (eg, “sit up straight”). When starting the workout, a 10-second countdown will be shown before the first exercise appears on the display (Figure 2C). Users can skip to the next exercise by touching the screen. They can pause or discontinue the workout at any time.

On the home screen, users can tap on the hexagon symbol in the upper right corner to navigate to settings, where they can find a list of all exercises included. Upon selecting one of the exercises within the list, the users are given the option to block the exercise from being included in the workouts or view the exercise video and description. In addition, they can find a link to the project website, receive comprehensive safety information for exercising, and obtain contact information.

Users can make changes to their personal profile by selecting their name in the top row to access the profile settings. Within the section “Sportliches” (English: sporty), users can edit the information on their available exercise equipment (Figure 2E) and physical capacity (Figure 2F). Changes made to this information influence the exercise selection within the workouts.

### Study Design

This pilot mixed methods block-randomized controlled feasibility trial with an allocation ratio of 1:1 for the intervention and waitlist control groups is conducted at the German Sport University Cologne (GSU), Germany. The trial was registered with the German Clinical Trials Register (DRKS00030370).

Participants will be recruited at the GSU and the SCI center at the BG (*Berufsgenossenschaft*; English: occupational insurance association) clinic Tübingen, Germany. Participants will be recruited at the GSU via the project newsletter, the project website, social media, and by contacting rehabilitation centers and spinal cord associations. The BG clinic in Tübingen will recruit among their patients.

**Figure 3:**
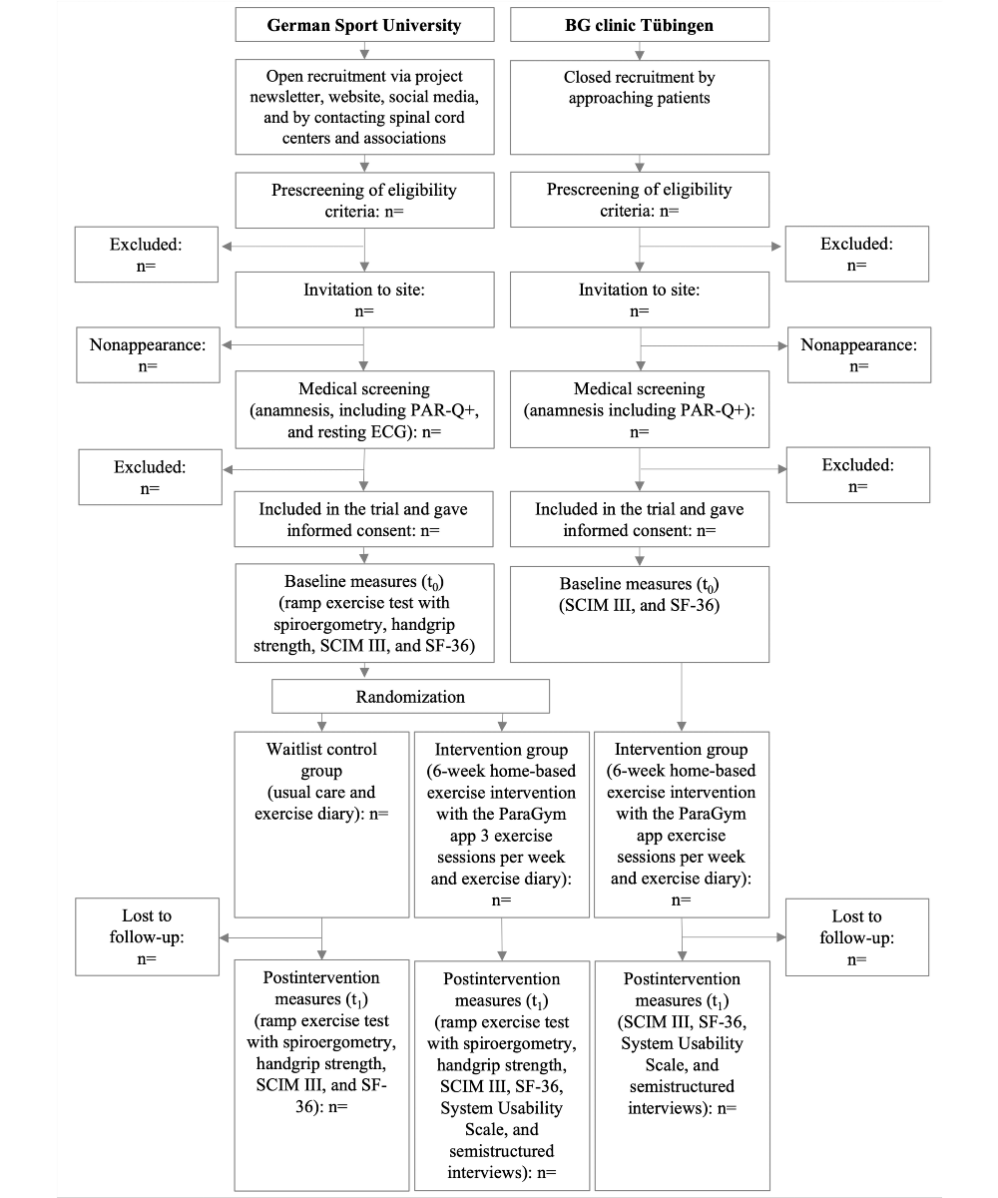
Study design and planned participant flowchart. BG: Berufsgenossenschaft; ECG: electrocardiogram; PAR-Q+: The Physical Activity Readiness Questionnaire for Everyone; SCIM III: Spinal Cord Independence Measure III; SF-36: Short Form–36 Health Survey.

Measurements will be conducted at baseline (t_0_) and after the intervention (t_1_; Table 1). The primary outcomes (feasibility, usability, and safety) will be assessed during the intervention and at t_1_. The secondary outcomes (peak exercise capacity, handgrip strength, independence, and quality of life) will be assessed at t_0_ and t_1_. As data collection at the BG clinic Tübingen will focus on the primary outcomes and owing to limited capacity, the ramp exercise test for evaluating peak exercise capacity and handgrip strength test will be conducted only at the GSU and not at the BG clinic Tübingen. After randomization and group allocation, participants in the intervention group will be provided with the ParaGym mobile exercise app and uptake a 6-week home-based training intervention. The participants will be instructed to complete 3 training sessions per week. For this cohort, resistance training for at least 2 days a week is recommended [2,6]. As the training sessions delivered by the app comprise not only strength but also aerobic and flexibility exercises, we decided to provide training sessions for 3 days instead of 2. Eitivipart et al [34] recommend 3 sessions for a minimum duration of 20 minutes per week over at least 6 weeks to achieve substantial outcomes. The waitlist control group will proceed with their usual care and receive access to the mobile app once they have completed the trial. After the 6-week intervention, participants in the intervention group will be interviewed over the phone to assess feasibility and usability. Subsequently, both groups will return to the study site (t_1_) for the SUS questionnaire and secondary outcome measures. The study design and planned participant flow are presented in Figure 3.

### Participants

#### Eligibility Criteria

Participants are eligible for the study if they meet the following inclusion criteria: (1) chronic paraplegia (time since injury >1 year), (2) age between 18 and 67 years, (3) sufficient German language skills, (4) ability to provide informed consent, and (5) access to a smartphone. Participants will be excluded from the trial if they have any of the following conditions or diseases: (1) acute infection or inflammation (eg, urinary tract infection or pressure ulcers), (2) uncontrolled hypertension or diabetes, (3) manifest cardiovascular diseases, (4) psychiatric or mental conditions that compromise informed consent or may interfere with the study results (eg, major depression and psychotic disorders), (5) ongoing alcohol or substance abuse, and (6) other medical contraindications. In addition, they will be excluded if they are high-performance athletes (in the national team).

Potential participants will be enrolled based on the results of the medical screening using their medical records, a resting electrocardiogram, and the Physical Activity Readiness Questionnaire for Everyone (PAR-Q+) [35]. Because participants recruited by the BG clinic Tübingen will not perform exercise tests, a resting electrocardiogram is not required.

#### Randomization

Only the 30 participants recruited at the GSU will be block randomized, stratified by the level (high vs low paraplegia) and completeness (complete vs incomplete paraplegia) of the SCI with a block size of 4. This will be done using Excel (Microsoft Corp). The randomization code will be released after the participant has completed all baseline measures. The randomization list will be password protected. Only JB and AL, who conduct the randomization and allocation, will have access to the list. No randomization is required at the BG clinic Tübingen, as data collection at that site focuses on the primary outcomes.

### Intervention

Following baseline measures, the intervention group will receive an introduction to the downloading process and a demonstration of the app [28,36-39]. They will also be provided with a user’s manual and 2 resistance bands with a resistance of their choice (TheraBand, The Hygenic Corporation), one of which is for backup. The intervention group will perform a 6-week home-based exercise program delivered by the ParaGym mobile exercise app, comprising three 35-minute exercise sessions per week. The app offers a different workout every day, and participants get to choose the days on which they want to exercise. An exercise session consists of 4 parts: warm-up, 2 exercise parts, and cooldown. The first exercise part focuses on the correct execution of the exercises at a moderate intensity and prepares the musculature for the high-intensity second part. Exercise sessions aim at various training goals, such as stabilization of the shoulder girdle and improvement of wheelchair mobility. The waitlist control group will only maintain usual care and receive access to the mobile app after postintervention measurements.

### Measures

#### Primary Outcomes

##### Overview

The primary outcomes are feasibility, usability, and safety. Six weeks after commencing the intervention, semistructured interviews will be conducted via phone to investigate feasibility and usability. An interview guide was conceptualized based on interview domains and topics in similar studies [27,36,39-43]. The following topics will be addressed: (1) overall experience, (2) ease of use, (3) app functionality, (4) perceived safety, (5) technical functionality, (6) communication, (7) perceived effects and usefulness, (8) adherence and motivation, and (9) recommendation. The interviews will be audio recorded with the consent of the participants.

##### Feasibility

During their first site visit, participants in both the intervention and waitlist control groups will be provided with an exercise diary (Multimedia Appendix 2). They will be asked to record the following: (1) any physical activities, including app exercise sessions, additional exercise sessions (eg, hand biking), and physical therapy; (2) the time spent performing the exercise; (3) the intensity of the exercise; and (4) any annotations concerning the exercise sessions as needed (eg, whether the session adapted by the algorithm was appropriate in terms of the choice of exercise and intensity). This will help obtain more information about the feasibility of the app in addition to the information provided in the retrospective interviews and help assess adherence to the program. Adherence rates in terms of completed sessions will also be measured based on app use data. Furthermore, retention rates will be measured by assessing dropout rates and reasons. As both adherence and retention are study outcomes, they should not be influenced by the investigators. Therefore, no measures will be taken to promote either of them. All outcome measures will be obtained regardless of adherence.

##### Usability

The SUS [44] is a 10-item Likert scale ranging from 1 (“strongly disagree”) to 5 (“strongly agree”) and is a common tool for investigating the usability of health-related mobile apps [27,36,38,39,41-43,45,46]. The score will be converted according to Brooke [44], so the total score will range from 0 to 100. The app will be considered usable if the overall SUS score is at least 70 [47].

##### Safety

The safety of the app-based exercise program will be determined based on the occurrence of intervention-related AEs and SAEs. Participants will be instructed to report any AEs or health-related interruption of the intervention within 24 hours. AEs and SAEs will be classified by the grade of severity based on the Spinal Adverse Events Severity System version 2 (SAVES-V2) scale [48] (Table 2) and expectance. The following AEs and SAEs can occur because of physical strain or are common complications after SCI and are, therefore, classified as expected AEs or SAEs: (1) autonomic dysreflexia, (2) pressure ulcers, and (3) shoulder pain [49].

**Table 2:** Classification of adverse events (AEs) and serious adverse events (SAEs) based on the Spinal Adverse Events Severity System version 2 scale.

Severity of AE or SAE	Clinical impact
1	AE—no treatment
2	AE—outpatient treatment
3	SAE—inpatient treatment
4	SAE—life-threatening injury
5	SAE—death

#### Secondary Outcomes

##### Overview

The secondary outcome is the effect of the intervention on peak exercise capacity, handgrip strength, independence, and quality of life. Peak exercise capacity and strength parameters were chosen because they are important factors for improving quality of life [11,15] and independence [10,14]. Quality of life and independence were chosen because these parameters are integral to the overall rehabilitation goal of people with SCI [50]. Furthermore, the guidelines for the conduct of clinical trials for SCI [51] suggest using the Spinal Cord Independence Measure III (SCIM III) for functional assessments and the Short Form–36 Health Survey (SF-36) for quality-of-life assessments.

##### Ramp Exercise Test

Participants will perform a ramp exercise test on an arm crank ergometer (ergo-metrics 800s, Ergoline). The rotational axis of the ergometer will be set at shoulder level. The distance between the wheelchair and ergometer will be set such that there is a slight flexion in the elbow joint when the participant extends their arm. A 3-minute warm-up will be conducted at a cadence of 60 to 70 revolutions per minute and a resistance of 0 W. After the warm-up, the workload will be increased by 10 W per minute (1 W every 6 seconds) until volitional exertion is reached [52]. Volitional exertion is reached when the cadence drops below 50 revolutions per minute [52] or rate of perceived exertion exceeds 18 on the Borg scale ranging from 6 to 20 [53]. Heart rate (Polar H7 chest belt, Polar Electro Oy), workload, and respiratory gas exchange parameters (MetaMax 3B, Cortex) will be monitored throughout the exercise test [52]. Participants will be asked to rate their level of perceived exertion on the Borg Rate of Perceived Exertion scale upon the termination of the exercise test.

##### Handgrip Strength

The determination of handgrip strength using a handgrip dynamometer is a widely applied, simple, and reliable method for evaluating the efficacy of a treatment [54] and predicts functional independence [55]. An electronic hand dynamometer (Trailite TL-LSC1000, LiteXpress GmbH) will be used to determine maximal handgrip strength. Participants will be asked to grip the handle with maximal effort [56]. As warm-up trials decrease the variability of strength measurements [54], participants will be given 1 warm-up trial for each hand. A total of 3 attempts will be made for each hand for a duration of 3 seconds each at a 90-degrees elbow flexion and neutral wrist position [54,55,57]. A rest period of 60 seconds will be provided in between. The sum of the highest values for the right and left hands will be used to determine handgrip strength [55].

##### Independence

The SCIM III was developed to sensitively detect changes in function (eg, concerning respiration) in individuals with SCI [58] and changes in various activities of daily living (eg, bed-wheelchair transfers) [59]. It shows a very high interrater reliability (*r*=0.98; *P*<.001). The SCIM III is subdivided into 3 categories: (1) self-care, (2) respiration and sphincter management, and (3) mobility. The score ranges from 0 to 100. A higher score indicates greater independence. In this study, the German version of the SCIM III will be used [60].

##### Quality of Life

The SF-36 is the most commonly used questionnaire to assess quality of life in people with SCI and has been shown to be valid and reliable for this population [61]. The survey comprises 36 items across 8 domains. The score ranges from 0 to 100, with a higher score indicating a better quality of life [62]. As previously recommended [11,63,64], 2 items referring to “climbing stairs” were replaced with “going up stairs” (German: *Stufen überwinden*), and 3 items referring to “walking” were replaced with “going” (German: *fortbewegen*).

### Data Management and Analyses

#### Data Management

Data will be entered into Microsoft Excel (version 2018) by one researcher and double-checked by another researcher before being analyzed using SPSS Statistics (version 29, IBM Corp). To ensure confidentiality, data will be recorded using the participants’ study ID instead of their names. All data forms will be stored electronically and protected by a password in addition to being stored in paper form and secured in a locker in a room with restricted access. Only the authors of this manuscript will have access to the full data set. The principal investigator (JB) will monitor AEs. The data will be deleted upon participants’ request.

#### Sample Size

The aim is to recruit a total of 45 participants between November 2022 and January 2023: 30 participants at the GSU and 15 participants at the BG clinic Tübingen. As this is a pilot trial, the sample size was based on previous studies with similar aims and methods [27,28] and recommendations for usability testing [65].

#### Qualitative Analysis

The interview audio recordings will be transcribed verbatim and analyzed using a qualitative content analysis approach according to Mayring [66] using MAXQDA (version 22, VERBI). Categories arising from the interview guide will be determined a priori. Two researchers will identify codes for the first 3 interviews and assign them to a category deductively. An inductive approach will be used to create additional categories and subcategories. Disagreements between researchers during the categorization process will be discussed until a consensus is reached. The interviews will be revised upon considering possible changes in the category system. More interviews will be analyzed until data saturation is achieved [40,45].

#### Quantitative Analysis

Quantitative data analysis will be performed using SPSS. All data will be tested for normal distribution using the Shapiro-Wilk test. Study participant characteristics will be presented descriptively by calculating means and SDs for normally distributed variables. Median values and IQR will be calculated for skewed continuous variables, and absolute and relative frequencies will be calculated for nominal variables. Group differences at baseline will be calculated for independent samples using a 2-tailed *t* test, Mann-Whitney *U* test, or chi-square test depending on the distribution and level of measurement. At t_1_, normally distributed continuous variables will be analyzed using a mixed analysis of variance model to test for significant effects for group and time. For skewed continuous variables, Wilcoxon and Mann-Whitney *U* tests will be conducted to test the differences between the groups and between t_0_ and t_1_. The significance level will be set at *P*<.05. Absolute and relative frequencies will be reported for adherence and retention.

Feasibility will be assumed if (1) at least 66% of the training sessions (equivalent to 2 out of 3 sessions per week) are completed [43] and (2) the retention rate is at least 75% [67]. Exercise diaries will be analyzed in terms of the frequency, duration, and intensity of the training sessions. Safety will be assessed by comparing the number of study-related AEs or SAEs in the intervention group with that in the waitlist control group [68].

Spearman correlations will be used to assess the association between performance and strength outcomes and SCIM III and SF-36 outcomes. Subgroup analyses might be conducted for lesion level (high vs low paraplegia) and age. Missing data will not be imputed.

### Ethics Approval

The conduct of the trial was approved by the ethics committee of the GSU (ethics committee number 180/2021). Comprehensive study information is sent to potential study participants before their first site visit to allow sufficient time to fully read and understand all the information. Study information includes details on the following: (1) general information and background; (2) eligibility criteria; (3) study aims and procedures; (4) participants’ rights, duties, and benefits; (5) potential risks; (6) data protection and management; (7) insurance; (8) funding; and (9) contact persons.

Participants will confirm that they have read, understood, and agreed to the terms of study participation by providing written informed consent before inclusion in the trial.

Precautions that will be taken to minimize the risk of cardiovascular events and AEs include the application of exclusion criteria, the conduct of a medical screening before enrollment, the distribution of a safety advice sheet, and integration of safety advice into the app.

To ensure confidentiality, we pseudonymized all participants’ data (refer to the *Data Management* section). Participants will receive compensation for travel expenses and the interpretation of their test results upon study completion. Any amendments to the protocol will be documented, and substantial changes will need to be approved by the ethics committee.

## Results

This trial is part of the FIT-IN^3^ (ParaGym) project, which is funded by the German Federal Ministry of Education and Research in September 2020 (funding number 16SV8572). The project started in September 2020 and is expected to be completed by August 2023. Recruitment for the trial began on November 1, 2022. A total of 12 participants were enrolled in the trial at the time of the submission of this manuscript (Table 3). Data collection will begin in January 2023 and is expected to be completed by April 2023. The results will be published in accordance with the CONSORT (Consolidated Standards of Reporting Trials) 2010 statement for randomized pilot and feasibility trials [69] as well as the CONSORT-EHEALTH (Consolidated Standards of Reporting Trials of Electronic and Mobile Health Applications and Online Telehealth) statement [70].

**Table 3:** Characteristics of the participants (n=12) enrolled in the study at the time of submission.

Characteristics			Intervention group (n=8)		Control group (n=4)
**Gender, n (%)**					
	Women	4 (50)		2 (50)	
	Men	4 (50)		2 (50)	
Age (years), median (IQR)			55 (37-62.5)		34 (18.5-56)
**Lesion level, n (%)**					
	T1-T6^a^	3 (38)		0 (0)	
	T7-S5^b^	5 (62)		4 (100)	
**Lesion completeness, n (%)**					
	Motor complete (AIS^c^ A-B)	6 (75)		2 (50)	
	Motor incomplete (AIS C-D)	2 (25)		2 (50)	
Time since injury (years), median (IQR)			11 (4.5-26.5)		17 (8-24.5)
**Experience with exercise apps, n (%)**					
	Yes	4 (50)		0 (0)	
	No	4 (50)		4 (100)	

^a^T: thoracic vertebra.

^b^S: sacral vertebra.

^c^AIS: American Spinal Injury Association Impairment Scale.

## Discussion

### Principal Findings

To the best of our knowledge, this is the first trial to investigate the feasibility, usability, and safety of a 6-week exercise program delivered by an intelligent mobile exercise app for individuals with paraplegia using qualitative and quantitative measures. As secondary outcomes, the preliminary effects of this program on peak exercise capacity, handgrip strength, independence, and quality of life will be assessed. As the app is currently at a prototype stage, technical issues compromising feasibility and usability might occur. Both the semistructured interviews and exercise diaries will provide detailed insights into the users’ experience with the app. This will help identify various aspects regarding the feasibility and usability of the exercise sessions, app’s interface, and app’s technical functionality, including intelligent personalization. Furthermore, users’ practical suggestions for improvement will contribute to drawing practical implications. These could include the following: (1) further adapting the app to meet the needs of the target group; (2) fixing technical issues; (3) improving the ease of use, (4) improving the design, intensity, and duration of the exercise sessions; and (5) improving the overall satisfaction with the app. Before integrating the exercises into the app, all exercises, including the exercise videos and descriptions, were tested and evaluated in terms of feasibility, usability, and comprehensibility by 10 workshop participants with paraplegia. Therefore, we expect these exercises to be feasible and usable. The program is expected to be safe, as a very low incidence of AEs has been reported during exercise trials with this cohort [49,71].

Similar mobile apps for promoting a healthy lifestyle in people with SCI have been shown to be feasible [27,28]. However, these apps were not declared to be at a prototype stage. In contrast to these already existing mobile apps, the ParaGym mobile exercise app delivers individualized training sessions without the need for exercise selection by the users or health care professionals. Thus, training sessions can be performed independently by people without specific knowledge of training and exercise. In fact, the most frequently mentioned suggestion for improvement of the WHEELS app [27] was related to personalization and better tailoring to the user’s needs and functional capabilities.

This trial will provide preliminary insights into the potential effects of the intervention on peak exercise capacity, handgrip strength, independence, and quality of life. Integrated exercises were designed to be appropriate for a beginner’s fitness level, so they will be feasible for most participants.

### Strengths and Limitations

Using the SPIRIT 13 Guidelines [31,72] for reporting the planned trial and the SCI trial guidelines [51,73] for designing as well as randomizing and controlling the trial improves its strength. In addition, the GSU cooperates with the BG clinic Tübingen to reach a larger sample size. Another strength is the compatibility of the app with various operating systems such as iOS (Apple Inc) and Android. However, smartphones should not be older than approximately 5 years to guarantee absolute functionality.

The ParaGym mobile app is currently at a prototype stage. Therefore, only basic functions and features are currently included. This could impact the variety of exercises, especially for users with severe impairments, which may decrease their motivation to use the app. The conclusiveness and generalizability of the results will be limited owing to the small sample size. In addition, only healthy adults with paraplegia were included in the study. Therefore, many individuals will be excluded from the study. Generalizability is also limited because the prevalence of cardiovascular diseases is high in individuals with SCI [74,75]. In addition, not restricting additional sports activities may have a major impact on the postintervention measurement results. Moreover, selecting exercises that are mainly suitable for beginners may compromise the effectiveness for intermediate users. Finally, all exercises will be performed using body weight or a resistance band, which will limit the user’s opportunity to increase intensity compared with using additional weights.

### Conclusions and Implications

By using both qualitative and quantitative measures, the results of this trial will generate a comprehensive understanding of the limitations and strengths of the ParaGym mobile exercise app as well as possible solutions to address the limitations. In the next step, in addition to fixing technical issues, the app should be adapted according to the findings of this study. This could include the expansion of the exercise pool and the addition of more equipment to increase variability and meet the needs of more advanced users. Hence, this could lead to an increased intensity of exercise sessions, which might result in greater effectiveness. Furthermore, the app interface might need to be adjusted to improve the ease of use. Finally, users may desire more features, such as the opportunity to design individual workouts or the integration of gamification to visualize the training process and promote adherence.

This pilot study will provide important results that need to be considered in the planning of future trials with an updated version of the app, which would aim for a larger sample size, longer intervention duration, and more diverse target group. As other individuals who use wheelchairs may also benefit from using the app, a requirement analysis addressing a broader target group should be conducted. Subsequently, a full-scale multicenter trial [73] including a broader target group, for example, people with acute SCI, tetraplegia, or other impairments, should be undertaken. In addition, by solely including healthy adults with SCI, a large proportion of the population was excluded. The prevalence of secondary diseases, such as cardiovascular diseases, is higher in individuals with SCI than in able-bodied people [74,75]. Therefore, investigators of future trials should consider including individuals with secondary diseases to assess the safety of the intervention for this group as well. Finally, future trials should include follow-up measures to investigate long-term motivation and adherence. In the long term, a fully marketable version of the ParaGym app should be implemented to make it publicly accessible.

An additional application of the ParaGym app could be its incorporation into workplace health programs, offering an exercise program for individuals who use wheelchairs and individuals with high levels of sedentary time at work.

A feasible, usable, and safe intelligent exercise app would increase access to personalized, independent, and evidence-based exercise training for this cohort and, in the future, for other individuals who use wheelchairs.

## Appendix

Multimedia Appendix 1 SPIRIT (Standard Protocol Items: Recommendations for Interventional Trials) 2013 checklist.

Multimedia Appendix 2 Exercise diary.

## Abbreviations

AE: adverse event
BG: Berufsgenossenschaft
CONSORT: Consolidated Standards of Reporting Trials
CONSORT-EHEALTH: Consolidated Standards of Reporting Trials of Electronic and Mobile Health Applications and Online Telehealth
GSU: German Sport University Cologne
PAR-Q+: Physical Activity Readiness Questionnaire for Everyone
SAE: serious adverse event
SAVES-V2: Spinal Adverse Events Severity System version 2
SCI: spinal cord injury
SCIM III: Spinal Cord Independence Measure III
SF-36: Short Form–36 Health Survey
SPIRIT: Standard Protocol Items: Recommendations for Interventional Trials
SUS: System Usability Scale

## Data Availability

The results of the trial will be published with open access in international journals after study completion. Additional anonymized information is available from the corresponding author upon request.
