# Supplementary material for: Feasibility, Usability, and Safety of ParaGym, an Intelligent Mobile Exercise App for Individuals With Paraplegia: Protocol for a Pilot Block-Randomized Controlled Trial
Source: JMIR Res Protoc. 2023 May 19;12:e45652. doi: 10.2196/45652 (PMC10238958; doi:10.2196/45652)
Supplement: Multimedia Appendix 2 [file resprot_v12i1e45652_app2.pdf]

ParaGym

Deutsche Sporthochschule Köln
German Sport University Cologne

Mein Aktivitätsprotokoll					Probanden-ID
**Datum**	**Meine Aktivität (z.B. Therapie, Handbike, ParaGym etc.)**	**Intensität mit der Borg-Skala**	**Dauer (min)**	**Notizen**	
